# Addressing Late-arriving Surgeons in Support of First-case On-time Starts

**DOI:** 10.1097/pq9.0000000000000784

**Published:** 2025-01-07

**Authors:** Jonathan B. Ida, Jamie H. Schechter, John Olmstead, Archana Menon, Mary Beth Iafelice, Amod Sawardekar, Olga Leavitt, Jennifer M. Lavin

**Affiliations:** From the * Department of Otolaryngology, Head and Neck Surgery, Northwestern University Feinberg School of Medicine, Chicago, Ill.; † Division of Pediatric Otolaryngology, Head and Neck Surgery, Ann & Robert H Lurie Children’s Hospital of Chicago, Chicago, Ill.; ‡ Surgical Services and Anesthesia, Ann & Robert H. Lurie Children’s Hospital of Chicago, Chicago, Ill.; § Surgical Services, Ann & Robert H. Lurie Children’s Hospital of Chicago, Chicago, Ill.; ¶ Department of Pediatric Anesthesiology, Phoenix Children’s Hospital, Phoenix, Ariz.; ‖ Department of Pediatric Anesthesiology, Ann & Robert H. Lurie Children’s Hospital, Chicago, Ill.

## Abstract

**Introduction:**

First-case on-time starts (FCOTS) is an established metric of perioperative efficiency, impacting global perioperative throughput. Late-arriving surgeons are a common cause of late operating room (OR) starts. This project reflects a quality improvement effort to reduce late surgeon arrivals by 30% for 24 months and improve FCOTS.

**Methods:**

A multidisciplinary perioperative leadership team developed clear expectations, including tracking, roles, review processes, and consequences. These were broadly communicated among stakeholders, and feedback was incorporated. A new same-day surgeon-to-surgeon feedback mechanism was instituted for late surgeon arrivals, allowing for surgeon feedback and reiteration of expectations. Results were prospectively tracked for 24 months before and following implementation.

**Results:**

Late surgeon arrivals decreased by 45%, from 23.6 to 13 per month for 24 months before and following implementation, respectively (*P* < 0.001). Balancing measures did not see increases for the same periods. FCOTS increased from 66% to 72% postimplementation (*P* < 0.001). Statistical process control P-charts demonstrated centerline shifts for both metrics.

**Conclusions:**

Development and communication of a clear framework of expectations, review, and consequences, with ongoing monitoring, clear performance expectations, and timely feedback, can reduce late surgeon arrival and improve FCOTS. Direct and timely communication provided immediate feedback to late surgeons and indicated reporting errors, providing more accurate data on late starts. Consistent policy enforcement is critical for credibility.

## INTRODUCTION

First-case on-time starts (FCOTS) is a well-established measure of efficiency in perioperative management.^1–3^ Multiple studies have shown various benefits of starting ORs on time, including but not limited to improved timeliness through the day, improved patient and surgeon satisfaction, reduced overtime staffing across the perioperative continuum with resultant cost savings, and improved team morale.^4^ The most commonly cited cause of late OR starts across multiple reports is late-arriving surgeons (LAS),^1,5,6^ as the surgeon is generally the final individual to see the patient before transfer into the operative suite. For a multitude of reasons, this has also proven to be one of the most challenging causes of late starts to address, resulting in institutions preferring to pursue improvement of other causes of late starts instead.^2,7^

Improving FCOTS has become a significant quality focus of healthcare institutions, with multiple approaches reported in the literature. Given the nature of FCOTS, its associated efficiencies, and the high-cost, high-revenue nature of perioperative operations,^8^ institutions can hardly afford to avoid this issue. Additionally, surgeon conduct can affect perioperative team morale,^9^ and the subsequent costs associated with staff turnover.^10,11^ Furthermore, perioperative teamwork has been linked to patient safety.^12^ Thus, it is clear that surgeon conduct, professionalism, and accountability have explicit and implicit effects on perioperative performance.

As part of a comprehensive approach to FCOTS, this study aimed to reduce the frequency of LAS by 30%, from a baseline average of 24 to 16 per month within 4 months, and sustain during a 2-year period, and evaluate the concomitant improvement in FCOTS during the study period, utilizing the Institute for Health Improvement model for quality improvement (QI) methodology.

## METHODS

### Study Setting

A 360-bed free-standing tertiary pediatric hospital with 21 primary operating rooms, 83 staff surgeons, multiple community providers across various specialties, 36 anesthesiologists, and 9 nurse anesthetists, performing more than 15,000 surgical procedures annually during the study period.

**Fig. 1.**
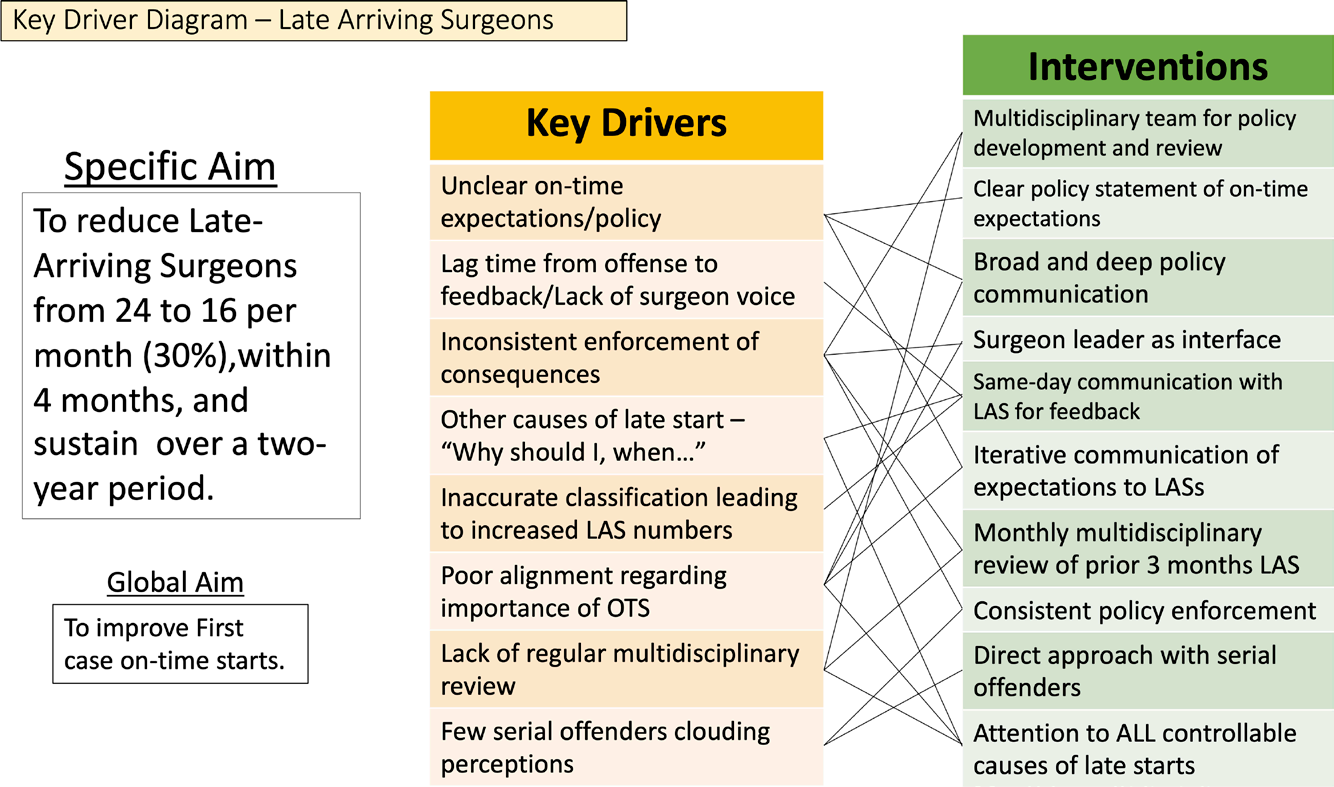
Key driver diagram noting significant potential drivers of poor surgeon compliance with on-time arrival and potential interventions suggested for remedies.

**Fig. 2.**
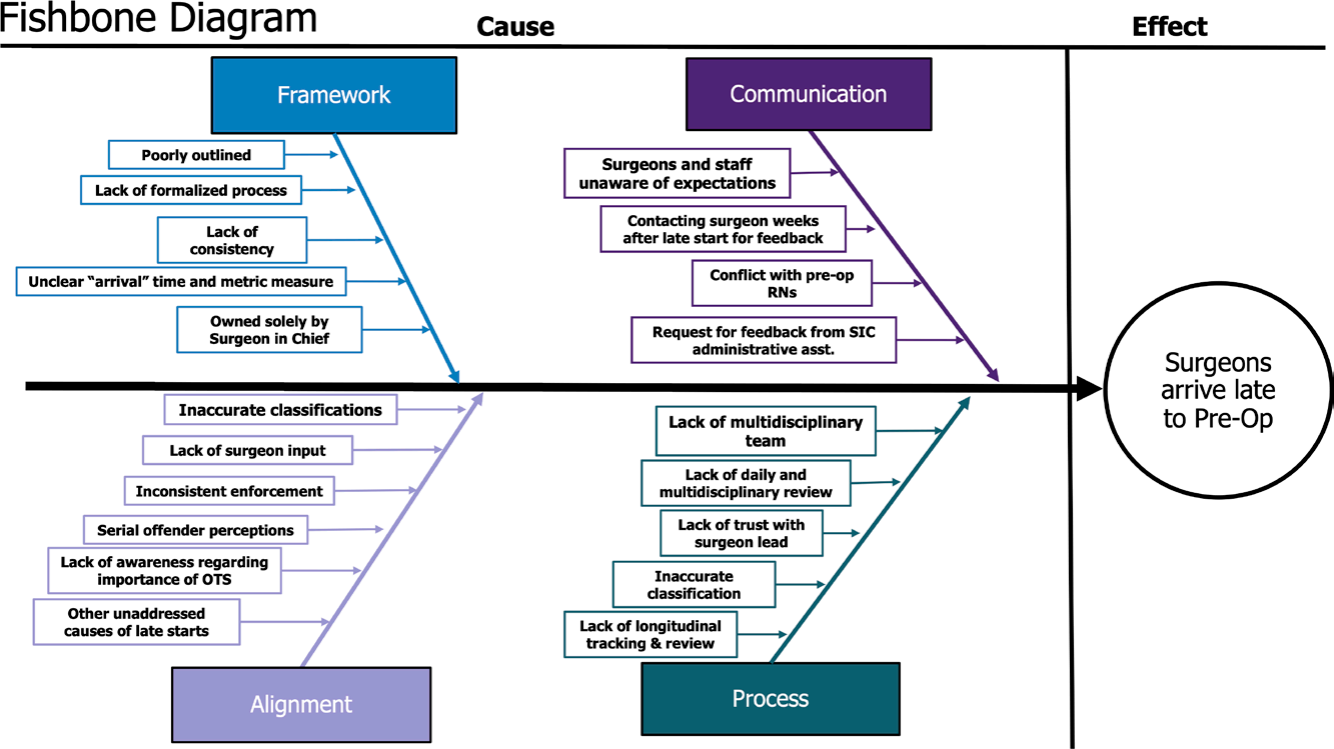
Fishbone diagram shows the improvement team’s impressions of causes of late surgeon arrival.

Surgeons at this institution are expected to arrive at preoperative no less than 15 minutes before surgical start time (ie, 715 for a 730 start), and OR starts are considered late if the patient enters the room more than 5 minutes after the scheduled start time (ie, 735 for a 730 start). The preoperative nurse (RN) documents late surgeon arrival. In the prior state, surgeons were contacted by the surgeon in chief’s office monthly regarding their late arrivals for the preceding month, and an explanation for each was requested. This meant individuals were contacted 4–6 weeks after the late arrival. If a surgeon was perceived as late consistently, they were subject to a potential adjustment to their block allocation. To the authors’ knowledge, this had been implemented once with significant unintended consequences (ie, late running ORs). Utilizing the Institute for Health Improvement “Model for Improvement” methodology, a key driver diagram (Fig. 1) and a fishbone diagram (Fig. 2) were developed for this project.

### Interventions

#### Establishing and Communicating Clear Expectations

**Fig. 3.**
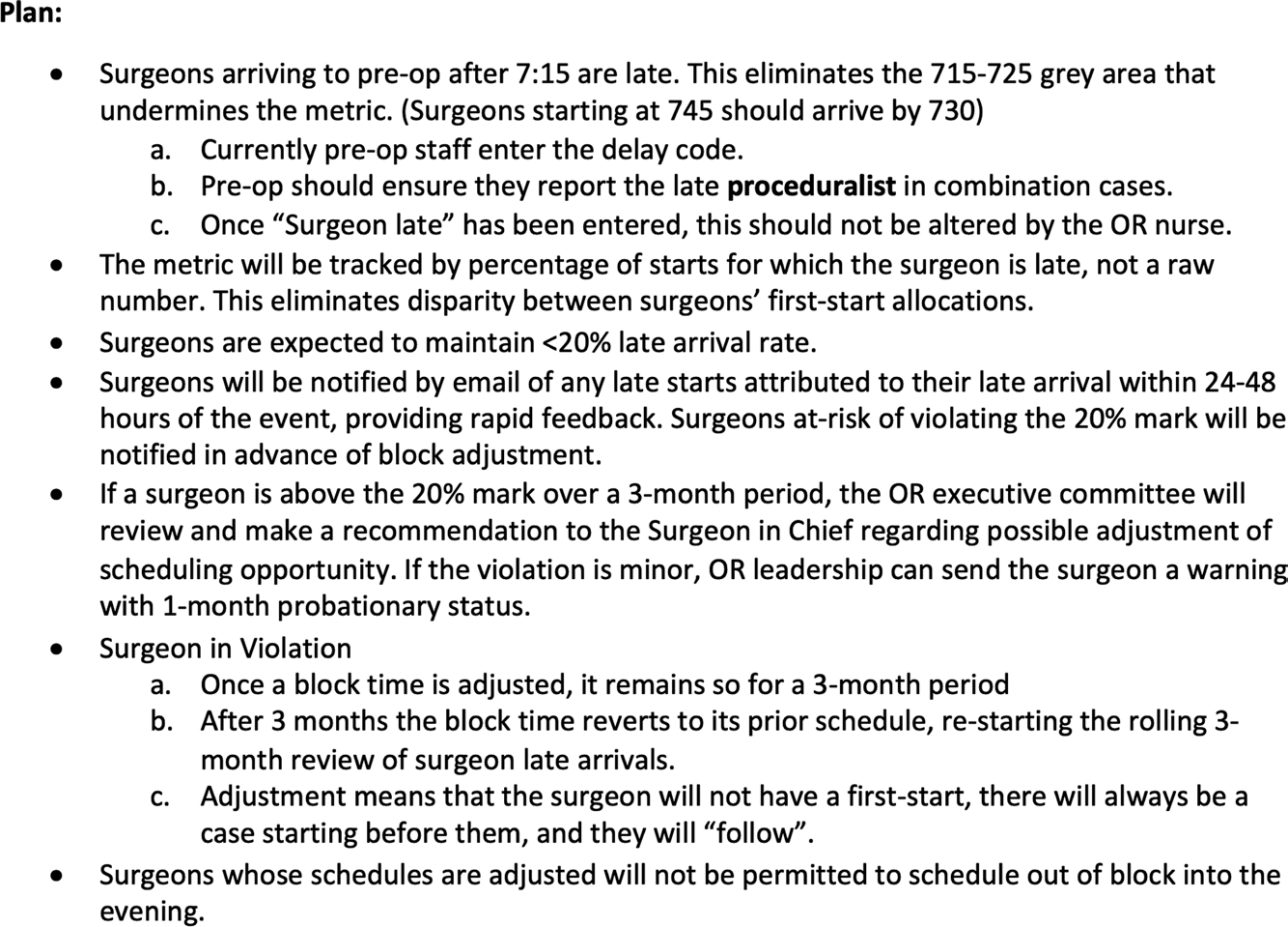
Policy outline developed by a multidisciplinary task force and delivered to all surgeons via multiple venues leading up to implementation.

To ensure that clear expectations were communicated broadly to the surgeons, a new framework was developed in anticipation of implementation. This framework included explicit expectations of time of arrival for surgeons and timing for other preoperative functions at the beginning of the day (ie, complete consent and verify readiness in the Electronic Medical Recored) and what will constitute a late start and an LAS (Fig. 3). These expectations were developed through a multidisciplinary team with representation from throughout the perioperative spectrum, including surgeons, anesthesiologists, nurses, and administrators, and communicated repeatedly and broadly to surgeons and proceduralists through various mechanisms, including emails, grand rounds, faculty meetings, departmental operational and quality/safety forums. This included direct conversations with 2 surgeons notorious for late arrivals regarding the upcoming implementation conducted by the surgeon lead for the improvement group. As a quality improvement initiative, this study was exempt from institutional review board review.

#### Reporting and Monitoring Late Starts

The cause of delay is entered by the preoperative RN assigned to the patient, often in collaboration with the OR RN, who is best positioned to identify and report these causes. As members of the improvement team, the preoperative RN manager and director reviewed these causes daily and reconciled discrepancies in collaboration with the surgeon lead. A daily FCOTS report is compiled and sent to a group of perioperative leaders before midday, including data on LAS. LAS is reviewed monthly by the multidisciplinary group in rolling 3-month increments.

#### Verifying the LAS and Reiteration of Expectations

Surgeons reported as arriving late were contacted by a peer-surgeon leader the same day to verify their late arrival or dispute this and provide another cause of the late start that the preoperative RN may not have recognized. The surgeon’s reason for the late start was recorded for later use in a review if needed. If LAS is verified, a form letter is sent to the surgeon reiterating the expectations and potential consequences of serial violation of expectations. The peer-surgeon leader was the department of surgery vice chair for perioperative operations, and a department member for over a decade at implementation.

#### Consequences

Surgeons may be late for no more than 20% of their first-case starts over any 3 months, or the multidisciplinary committee will take action, which could include notification and a probationary period or suspension of the surgeon’s access to first-case starts for a minimum of 3 months. A surgeon in violation would have to follow another surgeon. Although generous, the 20% cutoff was chosen because the institution lies in the center of one of the largest metropolitan areas in the United States, with inherent transportation challenges. Additionally, as the last person to see the patient before transfer to OR, there is always some expectation for the surgeon to be responsible for a late start.

#### Data Analysis

Data were prospectively collected for this study through the initiative’s first 2 years and compared with results from 2 years preimplementation. Two years was considered an adequate sampling for the long-term efficacy of a newly introduced system, demonstrated sustainability, and provided adequate comparison with the 2-year prior data. Balancing measures included monitoring for an increase in alternate reasons as surrogates for late surgeons: “patient/family initiated” and “medication delay” had both been noted in the past to increase with efforts to improve LAS. It was presumed that if a surgeon arrived late and a patient needed a premedication or the family had several questions, using these delay codes was considered nonconfrontational and nonpunitive.

This project utilized 2 statistical analyses, including a simple 2-sample *t* test and QI Statistical Process Control (SPC) methodology. Assuming an alpha of 5%, a 2-sample *t* test was performed to compare all variables pre- and postimplementation. QI methodology utilizing SPC p-charts was used for late-arriving surgeons and on-time starts. The numerator for the late-arriving surgeons was the number of first-case delays due to late surgeons, and the denominator was the total first cases. For on-time starts, the numerator was the number of first cases with no delays, and the denominator was the total number of first cases. The rule for centerline shift included 8 consecutive points above or below the previously established mean. Statistical analysis was performed using Microsoft Excel 2021 (Microsoft Corporation, Redmond, Wash.).

## RESULTS

**Fig. 4.**
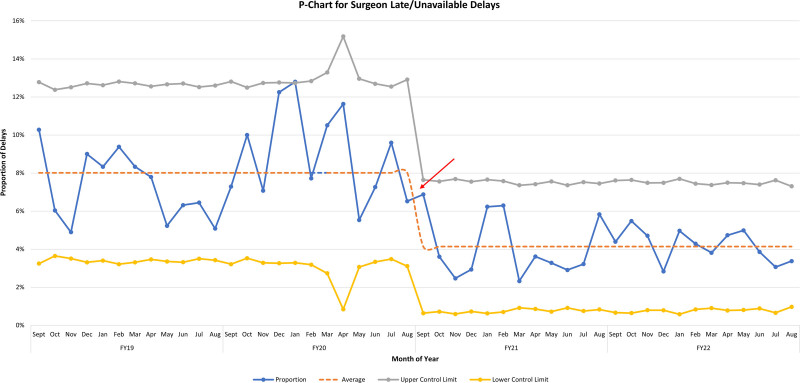
Statistical process control P-chart of documented LASs by month. Intervention noted September 2020.

**Fig. 5.**
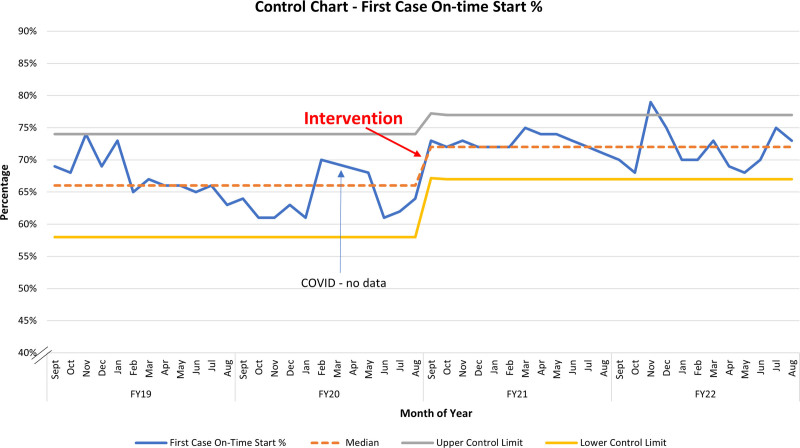
Statistical process control chart P-chart of FCOTS for 2 years pre- and postintervention.

**Fig. 6.**
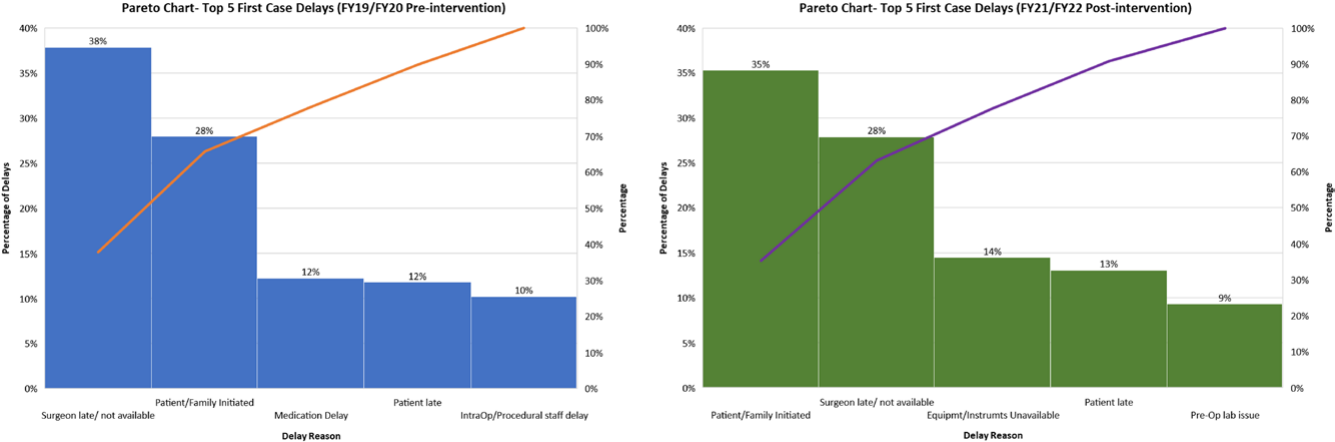
Pareto charts demonstrating changes in preintervention and postintervention top 5 late start reasons.

Implementation as described earlier on September 1, 2020, resulted in a near-immediate drop in late surgeon arrival. Over the 2-year study period, the frequency of late surgeons fell from a preimplementation rate of 23.6 incidents per month to 13 incidents per month, a 45% decrease (*P* < 0.001). The SPC signaled a centerline shift from 8% to 4% (Fig. 4). During the same period, first-case on-time starts increased from a preimplementation rate of 66% to 72% postimplementation (*P* < 0.001) and was consistently sustained through the 2-year study period. The SPC demonstrated a centerline shift soon after implementation (Fig. 5). Both SPCs show similarly sustained results throughout the study period. Balancing measures did not increase, with “patient/family initiated” showing a preimplementation frequency of 17.2 versus 16.3 per month postimplementation (*P* = 0.505) and “medication delay” showing rates of 7.4 and 8.8 per month, respectively, during the same periods (*P* = 0.115). Preintervention and postintervention Pareto charts (Fig. 6) showed a decrease in late surgeons by 10% as a proportion of the top 5 late start reasons and that late surgeons were no longer the most common cause of late starts. Total late starts decreased from 1,510 to 1,162 from preintervention to postintervention. Overall, late surgeons decreased from 571 to 324 between these 2 periods. Surgical volumes during the study period remained steady or increased with the exception of 2 months in 2020 due to COVID-19.

Multiple other data were reviewed by the study group for trends that would suggest opportunities for improvement, including daily and monthly case volumes, division-specific trends, and case types (ie, more complicated cases). The team did not identify any other opportunities. Although recording late arrival reasons provided by the surgeons, occasional opportunities arose. Most importantly, improved communication between the OR and division offices could result in better scheduling to match the surgeon’s academic or clinical schedule. These changes were implemented as they were identified.

During the study period, no surgeons required an adjustment of their block time due to late arrival above the limit. Among the 85 surgeons in the department, 2 underwent a review of their late arrivals and a warning with a probationary period during which performance improved to expectations. One surgeon underwent further discussion with escalation to the chair of the department of surgery to stress expectations, followed by a probationary period with good results. All 3 of these surgeons were mid-career, with 15+ years in practice, one is a private practice surgeon who operates exclusively at our institution. By the end of the study period, no other surgeons had required these discussions.

## DISCUSSION

Multiple disparate factors must align for an OR day to start on time: equipment must be processed and available, patients must arrive on time, perioperative staff must have the OR ready and the patient worked up, anesthesiology must review and process the patient, including any premedication, and the surgeon must be on time to see the patient and perform any further functions, such as site marking, presurgical discussion, and consent. In general, the surgeon interaction is the final stage of the workup process, and as the final step, represents a unique opportunity for causing delays. As such, late surgeons is a well-recognized cause of late starts in the OR and is frequently cited as a common contributor to poor FCOTS metrics.^3,5–7,13^

On-time starts have been addressed in multiple institutions with varying strategies. Wright et al^7^ described success with later OR start times with an on-time rate more than 90%; however, this strategy theoretically results in decreased productivity by increasing pre-OR nonoperative time and potentially fewer procedures. Furthermore, this approach sought to adjust the system to fit the surgeon and not seek improvement through behavior change. Most other strategies have included managing factors more directly under the institution’s control, such as equipment management, patient arrival times, and staffing, under the presumption that surgeon arrival, as an individual behavior, would be more challenging to change.^3^ Fezza and Palermo^13^ described improving FCOTS by addressing late surgeon arrival solely by providing a daily email to all first-case providers, reporting an increase in on-time starts from 24% to 80%; however, no other efforts to improve FCOTS were reported and the data specifically regarding late-arriving surgeons over the study period was not reported, in favor of overall on-time improvement rates. Pashankar et al^3^ included prior-day surgeon notification of start time as part of a comprehensive plan to improve on-time starts, but this study also did not report LAS rates or improvement. Phieffer et al^1^ noted that surgeons were major owners of delays in their OR improvement project but similarly did not report late-surgeon-specific data, instead reporting the overall on-time performance results. To our knowledge, this is the first study specifically focused on addressing surgeon arrival times to the OR, and utilizing QI methodology to do so.

More difficult to evaluate is the effect the lack of professionalism and accountability from surgeons has on the team dynamic within the perioperative space. Studies from other teams have demonstrated the multiple impacts of disruptive surgeon behavior on patient safety and team morale, including deterrence from surgical careers.^4,9^ Although healthcare has increasingly grappled with nursing and other staffing shortages, perioperative nursing has reported a disproportionately high shortage in recent years.^14^ During the study period, our institution, similar to all of healthcare, struggled with significant nursing turnover. In preparation for this implementation, concern was raised regarding preoperative RN/surgeon conflict over this initiative. Notably, supporting the preoperative RNs was one of the reasons that strong surgeon leadership was required for this project. Nursing turnover has been an exceptionally high cost for healthcare institutions in recent years, with 1 comprehensive literature review identifying costs ranging from $21,000 to $88,000 per nurse per year, with 3 of the studies reviewed citing decreased productivity as the most significant cost.^11^ The perioperative space can be responsible for 60%–70% of an institution’s revenue^15^; as such, decreased productivity would theoretically be exaggerated in this setting. Thus, although prior reports appropriately focus on the costs of delayed or added OR time or patient safety effects from poor perioperative teamwork, these likely underreport the financial impact of problems with surgeon accountability. Additionally, recently emerging personnel shortages in anesthesiology, particularly pediatric anesthesiology, could further underpin institutions’ need for improved perioperative teamwork and morale.^16,17^ Addressing individual behavior requires understanding the drivers of the behavior, as well as clear statements of expectations and the reasons for said expectations, consistent review of results, consistent feedback to the individual regarding problematic results, and fair and consistent implementation of consequences.

Regarding LAS and FCOTS, a broad approach to improving multiple factors that affect on-time starts would provide the best and most consistent results. The authors believe that LAS was an integral part of improving the overall process from a team morale and professionalism perspective, along with an opportunity to make a significant difference in FCOTS outcome metrics. Prior efforts at our institution were inconsistent, poorly timed, and poorly communicated, providing the leadership team an opportunity to give the surgeons clear and consistent goals and metrics, a voice in the process to ensure accuracy and accountability, and a consistent, same-day feedback mechanism with iterative messaging regarding expectations.

Multiple behavioral change theories exist and promote certain specific conditions to achieve individual behavior change: (1) an understanding of the problem and the effects perpetrated therein, (2) the individual’s role in the problem, (3) clear expectations and consequences, (4) consistent communication regarding results, and (5) consistent enforcement across the cohort. Social Cognitive Theory allows surgeons to self-evaluate their standing in the cohort, and most such individuals will self-adjust.^18^ In other words, when provided with the data, most people do not want to be negative “outliers” and will reflexively improve the unwanted behavior. Diffusion of Innovation Theory and the Fogg Behavior Model postulate that severe serial offenders (ie, laggards) would only respond to a perceived threat to their livelihood, reputation, or routine (ie, reduced OR time).^19,20^ In short, if being an outlier is not adequate motivation, some form of pressure can be applied, such as adjusting block time.

A key factor noted in this project included iterative communication of expectations after go-live, continuing to provide a consistent message to late surgeons, and an understanding that leadership is continuously monitoring this metric. This audit and feedback approach is a known and reliable QI technique that improves professional practice behaviors.^21^ This developed an institutional and departmental alignment on this metric. It was also critical to have a surgeon leader spearhead the initiative and function as the interface to the department, receiving and providing feedback to and from surgeons, ensuring that the process was not perceived as punitive, and continuing to message the work that was done in other areas in support of FCOTS. Finally, applying the policy equitably among all providers regardless of rank or status was critical to surgeon support for the process.

Within 2 months of implementing the framework described in this study, LAS decreased by nearly 50%, through a combination of direct appeals to known serial offenders, repeated and direct communication of results to surgeons from a peer-surgeon leader, and consistent enforcement across the surgeon cohort. Although addressing a small number of high-volume offenders was an important part of the project’s initiation, these 2–3 surgeons’ improvements cannot account for the change seen upon implementation. It was also feared preimplementation that late-arriving surgeon numbers would simply shift to another late start reason that would mask the metric, such as “patient and family initiated” or “medication delay,” and so these balancing measures were closely tracked and not observed. Additionally, FCOTS rates demonstrated a sustained improvement throughout a 2-year, prolonged study period, suggesting sustained improvement and not just a temporary or Hawthorne effect. The ability for a surgeon to dispute their late arrival on the same day certainly played a part in the categorization of late starts. However, given that before this effort the surgeons had little to no voice in the process, the management team felt that they had *more accurate* information regarding delays by including the surgeons in the assessment. Although this suggests that preimplementation, a certain percentage of LAS was incorrect, and the decrease from 24 to 13 per month was partially due to improved reporting accuracy, this only further reinforces the success of this project in improving the management team’s on-time starts data.

Since completing the study period, the workflow described above continues, with daily review of on-time metrics, including late-arriving surgeons, and quarterly improvement team meetings to review on-time starts. Workflows have been iteratively adjusted for streamlining, providing workflow sustainability for the lead surgeon on the team. Late-arriving surgeon numbers have been maintained since the study period, but ensuring continued compliance requires continued monitoring, albeit less intensively than during implementation. The work herein represents the application of behavior change principles organized by QI methodology and is therefore readily applicable and generalizable to multiple potential improvement or change management efforts.

## CONCLUSIONS

Late-arriving surgeons is a metric that can be successfully addressed with a carefully constructed plan to target drivers affecting behaviors. Decreased late surgeons and the resultant efficiencies are valuable to institutions, leading to measurable improvements in revenue and costs, and may lead to unmeasured gains in teamwork, morale, staff turnover, and safety. Improving late-arriving surgeon metrics hinges on a comprehensive approach and cannot be performed without addressing other factors that cause late starts, as collective surgeon alignment is critical to success. Conversely, comprehensive approaches to FCOTS should include a detailed plan to improve surgeons’ adherence to expectations.
